# Contraception use among individuals with substance use disorder increases tenfold with patient-centered, mobile services: a quasi-experimental study

**DOI:** 10.1186/s12954-023-00760-7

**Published:** 2023-03-06

**Authors:** Emily A. Hurley, Kathy Goggin, Kimberly Piña-Brugman, Janelle R. Noel-MacDonnell, Andrea Allen, Sarah Finocchario-Kessler, Melissa K. Miller

**Affiliations:** 1 Division of Health Services and Outcomes Research, Children’s Mercy Kansas City, 2401 Gillham Rd., Kansas City, MO 64108 USA; 2 Department of Pediatrics, University of Missouri-Kansas City School of Medicine, Kansas City, MO USA; 3 Department of Population Health, University of Kansas Medical Center, Kansas City, KS USA; 4 University of Missouri - Kansas City School of Pharmacy, Kansas City, MO USA; 5 Swope Health Services, Kansas City, MO USA; 6 Department of Family Medicine, University of Kansas Medical Center, Kansas City, KS USA; 7 Division of Emergency Medicine, Children’s Mercy Kansas City, Kansas City, MO USA

**Keywords:** Contraception, Reproductive justice, Substance use disorders, Recovery, Sexual health, Mobile unit

## Abstract

**Background:**

Individuals with substance use disorders (SUD) have disproportionately high rates of unintended pregnancy. Reducing harm associated with this risk and its biopsychosocial consequences requires evidence-based, non-coercive interventions that ensure access to contraception for individuals who choose to prevent pregnancy. We examined feasibility and impact of SexHealth Mobile, a mobile unit-based intervention that aimed to increase access to patient-centered contraceptive care for individuals in SUD recovery programs.

**Methods:**

We conducted a quasi-experimental study (enhanced usual care [EUC] followed by intervention) at three recovery centers with participants (*n* = 98) at risk for unintended pregnancy. EUC participants were offered printed information on community locations where they could access contraception care. SexHealth Mobile participants were offered same-day, onsite clinical consultation on a medical mobile unit and contraception if desired. The primary outcome was use of contraception (hormonal or intrauterine device) at one-month post-enrollment. Secondary outcomes were at two-weeks and three-months. Confidence in preventing unintended pregnancy, reasons for non-use of contraception at follow-up, and intervention feasibility were also assessed.

**Results:**

Participants (median age = 31, range 19–40) enrolled in the intervention period were almost 10 times more likely to be using contraception at one-month (51.5%) versus the those enrolled in the EUC period (5.4%) (unadjusted relative risk [URR] = 9.3 [95%CI: 2.3–37.1]; adjusted relative risk [ARR] = 9.8 [95%CI: 2.4–39.2]). Intervention participants were also more likely to be using contraception at 2-weeks (38.7% vs. 2.6%; URR = 14.3 [95%CI: 2.0–104.1]) and three-months (40.9% vs. 13.9%; URR = 2.9 [95% CI: 1.1–7.4]). EUC participants reported more barriers (cost, time) and less confidence in preventing unintended pregnancies. Mixed-methods feasibility data indicated high acceptability and feasible integration into recovery settings.

**Conclusions:**

Mobile contraceptive care based on principles of reproductive justice and harm reduction reduces access barriers, is feasible to implement in SUD recovery settings, and increases contraception use. Expanding interventions like SexHealth Mobile may help reduce harm from unintended pregnancies among individuals in SUD recovery.

*Trial Registration* NCT04227145.

## Introduction

Ensuring access to contraception is critical for populations at high risk for unintended pregnancies, particularly those in states with restricted abortion access. Women with a history of substance use disorders (SUD) have long suffered disproportionately high rates of unintended pregnancies. A nationally representative sample of women reported a 70% increased likelihood of unintended pregnancy among women with preconception illicit or recreational drug use, compared to those with no drug use [1]. In a study of women with opioid use disorder, nine in ten pregnancies were unintended, a rate two to three times higher than in the general population [2]. Over the last two decades, the proportion of pregnant women reporting non-prescription opioid use has quadrupled and the proportion reporting methamphetamine use has doubled [3, 4]. Further, overall SUD rates have risen in the U.S. since the onset of the COVID-19 pandemic [5].

Women with history of SUD often desire contraception but face access barriers [6–9]. They report cost, insurance and/or transportation challenges, and are overall less likely to have regular contact with primary or reproductive health care than women without SUD [8, 10]. Women with SUD  may also avoid contraception care-seeking due to experienced or anticipated stigma from providers [6, 8, 9, 11]. Further, they often lack accurate information about contraception options [7, 8] or may not prioritize seeking contraception when actively trying to obtain substances [8]. Addressing these barriers can help individuals with SUD gain more control over when and if they become pregnant, and avoid the often devastating health and psychosocial consequences associated with unintended pregnancies (e.g., losing child custody) [12–15].

Entry into recovery services is an opportune time to help individuals meet unmet contraception desires. While contraception access is important at all stages of SUD including recovery, women describe recovery initiation as a time of peak readiness to address broader health needs. Further, women initiating recovery generally state they prefer to plan pregnancies for a time when they have reached stable, long-term recovery [8, 16, 17]. Meeting contraception desires with long-acting reversible contraception (LARC) early in recovery can also provide longer-term protection for the 40–60% of women who return to use within one year of beginning treatment [18].

Although some studies have shown increased contraceptive use with SUD recovery-based interventions, they have had limitations [19]. Few interventions have fully demonstrated commitment to principals of *reproductive justice* (the right to maintain bodily autonomy, including choice over reproduction) and *harm reduction* (the right to access services that reduce harmful effects of substance use without judgement or pressure to change behavior) [8, 9, 20]. Some have included the controversial use of incentives or directive behavior change goals to encourage contraception use [21, 22]. Further, many have not been able to provide LARC and have offered only limited options [23]. Our formative research suggested women would find contraception services valuable and make use of them without incentives, given that the services: (1) maximize access to the full range of contraception options, (2) provide contraception free or at minimal cost, (3) offer non-judgmental, non-coercive patient-centered care, (4) are delivered by qualified, trusted providers, and (5) are supported and promoted by their peers [9].

We designed an intervention, *SexHealth Mobile*, to meet these community-derived priorities for people with SUD at risk of unintended pregnancy. *SexHealth Mobile* featured a medical mobile unit (MMU) equipped with a range of free contraception options and a reproductive health care provider who offered counseling and prescriptions. The intervention also trained outreach leaders at recovery centers to support free choice and providing accurate, non-coercive information on contraception options. This pilot study examines the feasibility of *SexHealth Mobile* and its impact on contraception use among woman in SUD recovery programs in Kansas City, Missouri.

## Methods

We conducted a quasi-experimental study with an interrupted time series design (i.e., enhanced usual care [EUC] followed by intervention). We compared contraception use one-month after enrollment among participants enrolled in the two different time periods. The study was approved by The Institutional Review Board at Children’s Mercy Kansas City.

### Setting and participants

*SexHealth Mobile* was implemented in partnership with a federally qualified health center (FHQC) in Kansas City that was already operating a MMU to provide basic health services at community locations with high need, including recovery centers. For this study, we selected three recovery centers that already partnered with the FHQC to receive periodic MMU visits for basic health services (e.g., seasonal vaccination, basic screenings, and treatment of non-emergency illness and injuries). The three recovery centers were providing services for clients with any type of SUD, most commonly polysubstance use disorders that included use of methamphetamines and/or opioids. Two centers were residential, serving individuals who had initiated recovery and were expected to not be using substances other than tobacco while in the program. The other was outpatient, serving individuals at various stages of recovery and active substance use. Prior to our intervention, the FHQC provided contraception and reproductive health services (free or discounted) at their main center, but not yet on the MMU.. Recovery centers did not routinely screen for reproductive health needs but would informally recommend community clinics (including the FHQC partner) for individuals interested in services.

Recovery center clients were eligible if they were: (1) aged 18–40; (2) screened positive for a lifetime history of problematic drinking or drug use according to CAGE-AID tool [24]; (3) able to become pregnant (assigned female sex at birth, pre-menopausal, not sterilized or diagnosed with infertility) but not currently pregnant; (4) not currently using an intrauterine device or hormonal contraception (i.e., oral contraceptive pill, transdermal patch, vaginal ring, injectable, subdermal implant); and (5) not previously enrolled in either phase of the study.

### Procedures

Study staff worked with recovery centers to share information about the study and visited each site periodically for eligibility screening and enrollment. Interested clients were screened individually by study staff and those eligible provided written informed consent. Site visits continued until the sample size target for each time period (*n* = 46) was reached or exceeded.

All participants completed a 15-min baseline questionnaire via REDCap that included demographic, substance use, and reproductive health history. For the EUC period (Aug–Nov. 2020), we created a printed information sheet that listed community locations where each recovery center would typically recommend clients contact to access contraception care. By formalizing this contact sheet and offering it to women after the baseline questionnaire on reproductive health needs, we “enhanced” recovery centers’ usual care procedures for reproductive health. In the *SexHealth Mobile* intervention period (April-Sept. 2021), participants were offered the opportunity to see a reproductive health care provider on the MMU and, if desired, obtain contraception on-site. Intervention period participants not interested in MMU visits were also offered EUC printed information sheets. Participants in both time periods completed a 5-min post-intervention survey as well as 2-week, 1-month, and 3-month follow-up phone assessments. Participants were compensated $20 for completion of the baseline/post-intervention survey, $15 each for 2-week and 1-month follow-ups, and $20 for the 3-month follow-up.

### SexHealth mobile intervention

*SexHealth Mobile* is grounded in formative research and centered on reproductive justice and harm reduction principles. The intervention featured “SexHealth Mobile Days” where the MMU, clinical staff, and study staff would visit each recovery center. In preparation, we worked with the FHQC leadership and clinical staff to integrate contraceptive services within the MMU. This included one nurse practitioner with experience in patient-centered contraception care for SUD patients, one care assistant, and materials required for on-site provision of contraceptive medications (e.g., pregnancy tests, needles, syringes). The nurse practitioner (author AA) specialized in women’s health, independently placed contraceptive devices, and had worked at the partner FHQC for seven years (including previously providing general health care on the MMU). We worked with recovery centers and FHQC staff to ensure services were provided in a way that was acceptable to potential patients, including arranging for pregnancy testing with urine collected by patients themselves within recovery center facilities.

On SexHealth Mobile Days, the provider offered contraceptive options on the MMU free of charge, including hormonal (i.e., pills, transdermal patch, vaginal ring, injection, subdermal implant) and non-hormonal (diaphragm, condoms). Participants who chose short-term hormonal contraception (pills, patch, ring, injection) were given dosage for three months of pregnancy prevention and instructions for refills/follow-up at the main FQHC. The MMU was not outfitted with a standard patient exam table that could be used for gynecological procedures, thus we could not offer same-day IUDs. However, participants interested in IUDs could speak to the provider on the MMU, schedule a facility-based IUD insertion appointment at the main FHQC with the same provider, and obtain a bridging method to protect against pregnancy until IUD insertion. Further, participants interested in sexual and reproductive health services beyond contraception were also able to speak with the MMU provider and schedule appropriate facility-based FHQC appointments. Study staff also offered free condoms and home pregnancy tests at the enrollment table for anyone at the recovery center. We conducted ten SexHealth Mobile Days before reaching our target recruitment in intervention period, with four visits at the larger residential center, two at the smaller residential center, and four at the outpatient center.

At each recovery center, 2–4 individuals who already occupied formal roles as trusted resources for clients (7 peer mentors, 3 social workers) were trained as “outreach leaders” to promote SexHealth Mobile Days and organize interested clients. Training covered basic principles of reproductive justice and harm reduction, contraception options, needs and challenges faced by individuals with SUD in preventing unintended pregnancies, and strategies for using client-centered and trauma-informed approaches when talking about contraception.

Outreach leaders and study staff stressed that all activities on SexHealth Mobile Days were completely voluntary, and people could obtain a clinic referral or meet with MMU providers and receive free contraception even if they chose not to enroll in the study, or were not eligible due to previous enrollment in the EUC period or other ineligibility criteria. Individuals could also enroll in the study and meet with MMU providers even if they were unsure or not interested in taking up contraception.

### Outcome measures

#### Feasibility

We assessed feasibility of *SexHealth Mobile* according to selected Bowen feasibility constructs [25]. Table 1 lists and defines constructs and measures of assessment, including participant survey items and study staff field notes.

**Table 1:** Assessment of *SexHealth Mobile* feasibility: constructs, indicators, and measures

Feasibility Construct [25]	Indicator	Measure
*Demand* *(To what extent is the intervention likely to be used?)*	Proportion of intervention participants interested in an MMU visit on a SexHealth Mobile Day	Baseline survey item^1^
	Proportion of intervention participants completing MMU visits who received on-site contraception	Post-intervention survey item^2^
	Number of additional MMU visits among individuals not eligible for the study (e.g., not within age range, participated in EUC period, already using contraception but interested in other methods)	Study staff field notes
	Number of individuals (participants and non-participants) who took free condoms and pregnancy tests; number of study participants reporting taking free condoms at MMU visit	Study staff field notes; post-intervention survey^1^
*Acceptability* *(How do stakeholders react to the intervention?)*	Intervention participants’ ratings of post-MMU visit satisfaction and likelihood of recommending to a friend	Post-intervention survey items^2^ Overall satisfaction: (4-point scale; range: “not satisfied at all” to “very satisfied”) Likelihood of recommending MMU (5-point scale from “extremely unlikely” to “extremely likely”)
	Intervention participants’ ratings of patient-centeredness of MMU provider	Post-intervention survey items^2^ *Person-Centered Contraceptive Counseling Survey* (PCCCS) [26]: using “top score” (an “excellent” rating on all four items)
	Intervention participants’ ratings of outreach leader support	Baseline survey item^3^ Level of agreement that outreach leader supported them in making their own decision (4-point from “strongly disagree” to “strongly agree)
	Recovery center leadership support and cooperation in facilitating implementation	Study staff field notes
	Proportion of EUC participants who reported they would have used the MMU if it had been available	Post-intervention survey item^4^
*Implementation* *(To what extent can the intervention be implemented as planned?)*	Outreach leader activity in intervention period	Study staff field notes
	Intervention participants’ self-reported interactions with outreach leaders	Baseline survey item^1^
	Acceptability of patient flow, volume, and wait time for all stakeholders	Study staff field notes
	Ability of MMU/provider to meet needs of patients	Study staff field notes
*Integration* *(To what extent can the intervention be integrated within an existing system?)*	Overall successes and challenges of integrating recovery and FHQC service systems, including personnel collaboration, scheduling, and facilitating pre-MMU patient procedures (e.g., paperwork, urine samples, implant insertion)	Study staff field notes

^1^Intervention participants only; ^2^Intervention participants completing MMU visits only; ^3^Intervention participants who reported interactions with Outreach Leaders only; ^4^Enhanced usual care participants only

#### Primary and secondary outcomes

The primary outcome was use of IUD or hormonal contraception (pills, patch, ring, contraception injection, subdermal implant) at one-month post-enrollment. Secondary outcomes were use at two-weeks and three-months. We also explored group differences in confidence in preventing unintended pregnancy at post-intervention and as a change from baseline to post-intervention (reported on a repeated 5-point scale ranging from “not at all confident” to “extremely confident”) as well as reasons for non-use at one-month (including reasons for not attending clinic visits, picking up prescriptions, and/or starting birth control).

### Data analysis

Descriptive analysis was carried out for all variables, with comparisons made between groups with Chi-Square or Fisher’s Exact tests for categorical variables as appropriate based on cell values, and two-sided independent t-test for continuous variables. We compared groups as intention-to-treat on the primary and secondary outcomes using Fisher’s Exact test and Poisson regression with robust standard errors (unadjusted and adjusted). The adjusted model included demographic factors known to influence contraception use or identified as having influence through a series of bivariate associations with contraception use, including recovery site (residential vs. outpatient), pregnancy intention (trying to avoid/wouldn’t mind avoiding vs. other), pregnancy history (ever been pregnant vs. not) and recency of substance use (defined as use within the three months prior to baseline vs. none). We analyzed the primary outcome again including participants who had missed the one-month assessment but reported a contraception injection (which protects for three months) at post-intervention or two weeks. Quantitative analyses were completed in SAS Version 9.4. (SAS Institute, Cary, NC). Field notes were analyzed with qualitative coding to identify facilitators and barriers according to Bowen’s feasibility constructs [25].

## Results

### Baseline demographics

**Fig. 1:**
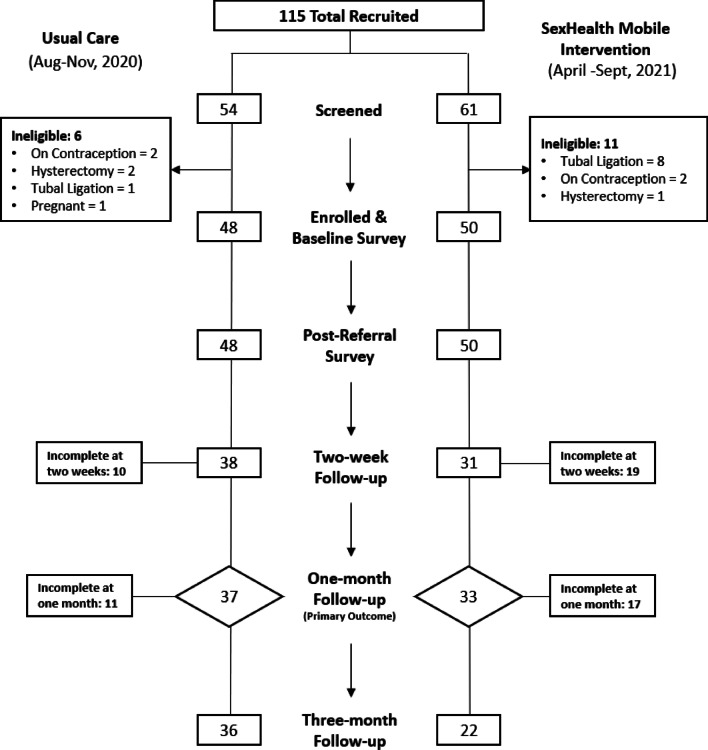
Screening, enrollment, & follow-up of participants in SexHealth Mobile Intervention

We enrolled 98 participants (48 in EUC period, 50 in intervention period). An additional 17 were ineligible after screening (Fig. 1). Follow-ups were completed by 70.4% at two-weeks (79.2% EUC, 62.0% intervention), 71.4% at one-month (77.1% EUC, 66.0% intervention), and 59.2% at three-months (75.0% EUC, 44.0% intervention). At baseline, participants in both groups were similar in age (median = 31, range 19–40), ethnicity (92.9% non-Hispanic), educational status (76.5% high school graduate or higher) and marital status (69.4% single/never married) (Table 2). Most identified as white (79.6%) with more identifying as Black/African American in EUC (12.5%) versus intervention periods (4.0%). One participant (in the intervention period) identified as male and all others identified as female. Almost half (46.9%) were uninsured while most others had public insurance (35.7%).

**Table 2:** Demographics (including substance use, sexual/repro health history)

	Enhanced Usual Care (EUC) (*n* = 48)	SexHealth Mobile Intervention (*n* = 50)	*p*-value
Age in years (Median, [IQR])	30 (25, 34)	32.5 (28, 35)	0.143
Race (*n*, %)			0.065
American Indian/Alaska Native	1 (2.1%)	1 (2.0%)	
Asian	0 (0.0%)	1 (2.0%)	
Black/ African American	6 (12.5%)	2 (4.0%)	
White	40 (83.3%)	38 (76.0%)	
Other	1 (2.1%)	7 (14.0%)	
Ethnicity			1.000
Non-Hispanic	45 (93.8%)	46 (92.0%)	
Hispanic/ Latina	3 (6.3%)	3 (6.0%)	
Educational Level			0.110
Less than High School	10 (20.8%)	13 (26.0%)	
High school graduate or GED	22 (45.8%)	24 (48.0%)	
Post-high school training/ some college	15 (31.1%)	9 (18.0%)	
Undergraduate degree or Higher	1 (2.1%)	4 (8.0%)	
Marital status			0.714
Single (never married)	34 (70.8%)	34 (68.0%)	
Separated/ Divorced	11 (22.9%)	11 (22.0%)	
Married/ domestic partnership	2 (4.2%)	3 (6.0%)	
Widowed	1 (2.1%)	2 (4.0%)	
Health Insurance Status			0.632
Private	2 (4.2%)	5 (10.0%)	
Public (Medicare, Medicaid)	16 (33.3%)	19 (38.0%)	
Other	3 (6.3%)	4 (8.0%)	
Uninsured	26 (54.2%)	20 (40.0%)	

^*^Where options do not add to 100%, one or more participants marked “prefer not to answer”

EUC and intervention participants were also similar in substance use and sexual/reproductive health history (*p*-values > 0.05 unless otherwise noted). Participants were receiving recovery services at the outpatient (48.0%) or one of two residential (52.0%) centers. Most participants had used substances other than tobacco within the past three months, with a greater proportion of EUC participants reporting recent substance use compared to intervention participants (85.4%, 66.0%; *p* = 0.026). The majority had used amphetamines (78.6%), cannabis (80.6%), alcohol (76.5%), opioids (59.2%), and/or cocaine (60.2%) at some point in their life. Most had a history of polysubstance use (i.e., use of two or more substances other than tobacco), with slightly more of the EUC (58.3%) than the intervention group (42.0%). Most (72.4%) indicated they had received some medical care in the past 12 months and 58.2% said there was a time in the past 12 months they thought they should have accessed health care but did not.

Most had no (43.9%) or one (44.9%) current male sexual partner with few reporting they were trying to get pregnant (2.0%). At last vaginal sex, most reported no pregnancy prevention method (48.0%) or withdrawal (24.5%). Four-fifths had not used a condom at last sex. The majority had lifetime experience with contraception, most commonly the pill (67.3%) and condoms (67.3%). Most had experienced pregnancy (80.6%) and two reported previous abortion. Most (72.4%) had at least one living child and of those with children, and 60.5% reported their child/children living in foster care or with someone else at some point.

### Intervention feasibility

Table 3 summarizes mixed-methods feasibility findings. Overall intervention *demand* was high, as 43 intervention participants (86.0%) requested an MMU visit for contraception counseling. Of the 39 who completed a visit and post-intervention survey, 22 (56.4%) reported receiving a form of hormonal contraception on the MMU (pills = 14, contraception injection = 5, subdermal implant = 1, patches = 1, rings = 1). Additionally, two participants (5.1%) were prescribed a diaphragm and 18 (46.2%) took condoms *Acceptability* was high, as most intervention participants who visited the MMU were “very satisfied” with their visit (92.3%) and gave the provider a top score for person-centeredness (86.8%). Observations and field notes indicated successful implementation and integration of services, with minor challenges (detailed in Table 3).

**Table 3:** *SexHealth Mobile* feasibility: Key findings

Feasibility Construct^1^	Facilitators ( +) and Barriers (-)
*Demand (To what extent is the intervention likely to be used?)*	86% of intervention participants indicated interest in an MMU visit on a SexHealth Mobile Day ( +)
	56.4% of intervention participants who visited the MMU received on-site contraception ( +)
	21 individuals not enrolled in the study had an MMU visit ( +)
	42 individuals took free condoms and 30 took free pregnancy tests from study staff; 18 study participants took free condoms at an MMU visit ( +)
*Acceptability (How do stakeholders react to the intervention?) *	92.3% of intervention participants who visited the MMU were “very satisfied” with their visit; 97.4% were “likely” or “extremely likely” to recommend to a friend (+)
	86.8% of intervention participants who visited the MMU gave the provider a top score for patient-centeredness ( +)
	76.6% of intervention participants who spoke to an outreach leader “agreed” or “strongly agreed” that the leader supported them in making their own decisions ( +)
	Recovery center leadership actively supported implementation at all three sites ( +)
	81.3% of EUC participants reported they would have used the MMU if it had been available ( +)
*Implementation (To what extent can the intervention be implemented as planned?)*	Outreach leaders successfully mobilized attendance for SexHealth Mobile Days ( +)
	60.0% of intervention participants reported having spoken to an outreach leader about SexHealth Mobile ( +)
	Patients with sexual or reproductive health needs beyond available contraception methods were able to have a preliminary consultation on the MMU and schedule a later appointment at the main FQHC ( +)
	Interest in MMU appointments (which were often lengthy) sometimes exceeded the number of interested individuals (-)
	Some women were asked to wait for a future confirmatory negative pregnancy test before receiving a subdermal implant (-)
*Integration (To what extent can the intervention be integrated within an existing system?)*	Pre-existing FQHC/recovery center relationships facilitated smooth service integration and service provision for patients regardless of insurance status ( +)
	MMU process were easily adapted to accommodate contraception care (including implant insertion and self-collection of urine) and SexHealth Mobile Days were easily integrated into recovery center activities ( +)
	Limited availability of the FQHC MMU and staff created scheduling challenges ( +)
	Patients not yet registered to receive services with the FQHC faced a high burden of paperwork (-)

^1^Full description of each construct’s operationalization and measurement is available in Table 1

### Contraception use

For our primary outcome, 51.5% (17/33) of participants in the intervention period were using contraception at one-month post-baseline compared to 5.4% (2/37) in the EUC period (Fishers exact test *p* = 0.001) (unadjusted relative risk (URR) = 9.3 [95%CI: 2.3–37.1]; *p* = 0.0016). Both EUC participants reported contraception injection, while intervention participants reported pills (*n* = 11), contraception injections (*n* = 3), implant (*n* = 1), patch (*n* = 1) and ring (*n* = 1). The proportion of intervention participants using contraception was also higher than that in EUC at 2-weeks (38.7% [12/31] vs. 2.6% [1/38]; *p* =  < 0.0001) (URR = 14.3 [95% CI: 2.0–104.1]; *p* = 0.0085) and three months (40.9% [9/22] vs. 13.9% [5/36]; *p* = 0.020) (URR = 2.9 [95%CI: 1.1–7.4]; *p* = 0.031).

Likelihood of contraception use at one-month remained high after adjusting for age, recovery center type, pregnancy intention, pregnancy history, and substance use at three-months (adjusted relative risk (ARR) = 9.8 [95%CI: 2.4–39.2]; *p* = 0.0013). One additional participant could be assumed to be using contraception at one-month despite not completing the assessment, as she reported receiving a contraception injection on the post-intervention survey, strengthening the relative risk in the unadjusted (RR = 9.7 [95%CI: 2.5–39.1]; *p* = 0.0012) and adjusted models (ARR = 10.0 [95%CI: 2.5–40.0]; *p* = 0.0012).

### Confidence and barriers

Self-reported confidence in one’s own ability to protect against unwanted pregnancy (dichotomized as “very/extremely confident” and “not confident/low confident/not sure”) was more prevalent in the intervention than the EUC group in the post-intervention survey (90% vs. 70%; *p* =  < 0.0001). A greater proportion of participants increased from “not confident/low confident/not sure” at baseline to “very confident/extremely confident” post-intervention in the intervention (28.0% ) versus the EUC group (12.5%; *p* =  < 0.001).

The most frequently cited reason for non-use of contraception at one-month among both groups was “decided did not want/need birth control” (EUC = 27.1%; intervention = 16.0%; *p* = 0.18). Some participants offered additional explanation for not being interested in contraception, including not being sexually active or not having male partners. A few also expressed ambivalence toward becoming pregnant. The barrier “not enough time” (to complete referral appointment, pick up prescription, and/or start method) was reported more frequently in the EUC group (22.9%) than the intervention group (6.0%; *p* = 0.001). “Insurance/cost” barriers were also reported more frequently in the EUC (14.6%) versus intervention (2.1%; *p* = 0.023). Less frequently cited barriers were “transportation” (EUC = 6.3%; intervention = 2.0%, *p* = 0.288) and “COVID-19-related” (EUC = 2.1%; intervention = 0%, *p* = 0.305).

## Discussion

Participants offered free, autonomy-supportive, mobile contraceptive services at SUD recovery centers were over nine times more likely to be using contraception at one-month post baseline than those exposed to EUC (51.5% vs. 5.4%). The estimated advantage of the intervention over EUC strengthens to tenfold when controlling for age, recovery center type, pregnancy intention, pregnancy history, substance use within three months of the study and assuming continual protection for the one lost-to-follow-up participant who received a contraception injection during her visit. Participants exposed to the intervention and not using contraception at one-month typically reported that this was their preference (they decided they did not want or did not need contraception) indicating that this was not a limit of the intervention, but rather a personal choice. Among those not using contraception at one-month, EUC participants reported significantly more barriers in terms of time and cost than intervention participants. Participants exposed to *SexHealth Mobile* also reported higher post-intervention confidence in their ability to protect against unwanted pregnancy.

Our intervention was designed to address key limitations of past interventions aimed at increasing contraception access for individuals with SUD. Based on formative research with our target community (interviews, focus groups) [9], we carefully designed all aspects of the intervention to stress reproductive justice (e.g., outreach leader role/training stressed a non-coercive approach, participation/enrollment for all regardless of contraceptive interest, and provision of all contraception options that could be accommodated on the MMU free of charge). Prior contraception access interventions for individuals with SUD have included behavioral strategies or incentives [19] and some have drawn concern about the potential for coercion from providers [21, 22]. Thoughtful recent adaptions have linked financial incentives to attendance at contraception appointments instead of to contraception use directly [27, 28], but this strategy was viewed as unnecessary and potentially problematic by our community partners. *SexHealth Mobile* was highly successful in increasing contraception use without the use of incentives. It also offered a sustainable intervention model by building off an existing FQHC and its trusted partnerships with local recovery centers. Our work also confirms feasibility of administering longer-acting contraception (injections and subdermal implant) via MMU in a U.S. urban setting [29, 30]. Though MMU-based IUD insertion is possible, [31] it was not feasible to offer through our intervention, and more research is needed to determine how to best integrate IUD provision into scalable MMU programs. Finally, while MMUs have shown high reach and uptake for contraception in low and middle-income countries, their use to among at-risk populations in the U.S. has not been extensively evaluated [31, 32]. Ours study is the first to estimate the impact of MMU-based contraception care compared to EUC.

*SexHealth Mobile* demonstrated high demand and acceptability, with strengths and limitations in its implementation and integration with existing services. The vast majority of intervention participants made use of the MMU, with high ratings of overall satisfaction and person-centeredness of the provider. Many intervention participants chose contraception (56.4%), but others reported they decided they did not want or did not need contraception, a strong indicator that they felt reproductive autonomy, and were not coerced toward contraception even if they elected to visit the MMU. Given our formative research findings that LARCs would be highly desirable option, the low uptake of subdermal implants on the MMU was surprising. On SexHealth Mobile Days, many participants were still hesitant or unfamiliar with subdermal implants and not ready to commit to the procedure. This may have been because many participants took advantage of MMU visits who had not been reached by education efforts leading up to SexHealth Mobile Days (40% reported not having prior conversations about contraception with an outreach leader) and thus may not have had adequate time to thoroughly consider new methods. Many expressed interest interested in learning about new methods like implants and considering them for a later date, but on a day-of, walk-in MMU service, were ultimately most comfortable obtaining familiar methods (e.g., pills). Another key barrier to implementation was that some participants were told to wait several weeks to conduct another pregnancy test before getting a subdermal implant (a standard practice at this FHQC but inconsistent with evidence-based guidelines) [33] and were given a bridge method until a later visit. Further, challenges in scheduling the MMU and paperwork for new patients sometimes limited the number of MMU visits offered within on SexHealth Mobile Day.

### Limitations

In this quasi-experimental design, participants were not randomized to conditions. The similarities in both groups on demographic factors proximally associated with contraception use, and the fact that all were recruited from the same sites, however, minimizes the possibility that differences observed between intervention/EUC conditions could be attributed to baseline participant characteristics. The EUC and intervention periods also occurred at different time points, thus there may have been inherent benefits to the intervention period occurring later (e.g., more exposure at each site to sexual health information/activities related to the study). We do not believe that the trajectory of the COVID-19 pandemic had a significant impact on results, as we did not initiate enrollment in the EUC period until in-person activities at recovery centers and referral health centers had resumed. In reporting barriers to contraception, just one participant in the EUC period named a COVID-19 related factor. We also note limitations in diversity in our sample as the majority of our sample identified as white and non-Hispanic.

## Conclusion

*SexHealth Mobile* expanded access to patient-centered contraception options for individuals with  SUD, demonstrating meaningful increases in contraception coverage and confidence to protect against unintended pregnancy, without the need for incentives or persuasion. Alleviating barriers to contraception by expanding interventions like *SexHealth Mobile* could help empower more individuals entering substance use recovery to prevent unintended pregnancy. More research is needed to adapt and implement similar interventions for individuals with SUD who are not connected to traditional recovery centers.

## Data Availability

The datasets used during the current study are available from the corresponding author on reasonable request.

## References

[1] Shafique S, Umer A, Innes KE, Rudisill TM, Fang W, Cottrell L (2021). Preconception substance use and risk of unintended pregnancy: pregnancy risk assessment monitoring system 2016–17. J Addict Med..

[2] Heil SH, Jones HE, Arria A (2011). Unintended pregnancy in opioid-abusing women. J Subst Abus Treat.

[3] Haight SC, Ko JY, Van Tong VT, Bohm MK, Callaghan WM (2018). Opioid use disorder documented at delivery hospitalization—United States, 1999–2014. Morb Mortal Wkly Rep.

[4] Admon LK, Bart G, Kozhimannil KB, Richardson CR, Dalton VK, Winkelman TNA (2019). Amphetamine-And opioid-affected births: incidence, outcomes, and costs, United States 2004–2015. Am J Public Health..

[5] Czeisler MÉ, Lane RI, Petrosky E, et al. Mental health, substance use, and suicidal ideation during the COVID-19 pandemic—United States, June 24–30, 2020. MMWR Morb Mortal Wkly Rep. 2020. 10.15585/mmwr.mm6932a1 10.15585/mmwr.mm6932a1 PMC7440121 32790653

[6] MacAfee LK, Harfmann RF, Cannon LM (2020). Substance use treatment patient and provider perspectives on accessing sexual and reproductive health services: barriers, facilitators, and the need for integration of care. Subst Use Misuse.

[7] Fischbein RL, Lanese BG, Falletta L, Hamilton K, King JA, Kenne DR (2018). Pregnant or recently pregnant opioid users: contraception decisions, perceptions and preferences. Contracept Reprod Med.

[8] Stancil SL, Miller MK, Duello A (2021). Long-acting reversible contraceptives (LARCs) as harm reduction: a qualitative study exploring views of women with histories of opioid misuse. Harm Reduct J.

[9] Hurley EA, Duello A, Finocchario-Kessler S (2020). Expanding contraception access for women with opioid-use disorder: a qualitative study of opportunities and challenges. Am J Heal Promot.

[10] Drescher-Burke K (2014). Contraceptive risk-taking among substance-using women. Qual Soc Work.

[11] McCartin M, Cannon LM, Harfmann RF, Dalton VK, MacAfee LK, Kusunoki Y (2022). Stigma and reproductive health service access among women in treatment for substance use disorder. Women’s Heal Issues.

[12] Lamy S, Laqueille X, Thibaut F (2015). Consequences of tobacco, cocaine and cannabis consumption during pregnancy on the pregnancy itself, on the newborn and on child development: a review. Encephale.

[13] Perez FA, Blythe S, Wouldes T, McNamara K, Black KI, Oei JL (2022). Prenatal methamphetamine—impact on the mother and child—a review. Addiction.

[14] Neonatal KP, Syndrome A (2014). Pediatrics.

[15] Scott LF, Shieh C, Umoren RA, Conard T (2017). Care Experiences of Women Who Used Opioids and Experienced Fetal or Infant Loss. JOGNN - J Obstet Gynecol Neonatal Nurs.

[16] Olsen A, Banwell C, Madden A (2014). Contraception, punishment and women who use drugs. BMC Womens Health.

[17] Hurley EA, Piña K, Cegielski V, Noel-MacDonnell JR, Miller MK (2021). Recovering from substance use disorders during the early months of the COVID-19 pandemic: A mixed-methods longitudinal study of women in Kansas City. J Subst Abuse Treat..

[18] McLellan AT, Lewis DC, O’Brien CP, Kleber HD (2000). Drug dependence, a chronic medical illness implications for treatment, insurance, and outcomes evaluation. J Am Med Assoc.

[19] Heil SH, Melbostad HS, Rey CN (2019). Innovative approaches to reduce unintended pregnancy and improve access to contraception among women who use opioids. Prev Med.

[20] Cadena DS, Chaudhri A, Scott C (2022). Contraceptive care using reproductive justice principles: beyond access. Am J Public Health.

[21] Lucke JC, Hall WD (2012). Under what conditions is it ethical to offer incentives to encourage drug-using women to use long-acting forms of contraception?. Addiction.

[22] Lyerly AD (2021). Beyond voluntariness—ethics and incentives for contraception. JAMA Psychiat.

[23] Yermachenko A, Massari V, Azria E (2020). Unintended pregnancy prevention in women using psychoactive substances: a systematic review. Addict Behav.

[24] Brown RL, Rounds LA. Conjoint screening questionnaires for alcohol and other drug abuse: criterion validity in a primary care practice. Wis Med J. 1995. 7778330

[25] Bowen DJ, Kreuter M, Spring B (2009). How we design feasibility studies. Am J Prev Med.

[26] Dehlendorf C, Henderson JT, Vittinghoff E, Steinauer J, Hessler D (2018). Development of a patient-reported measure of the interpersonal quality of family planning care. Contraception.

[27] Heil SH, Hand DJ, Sigmon SC, Badger MS, Meyer MC, Higgens ST (2016). Using behavioral economic theory to increase use of effective contraceptives among opioid-maintained women at risk of unintended pregnancy. Prev Med.

[28] Heil SH, Melbostad HS, Matusiewicz AK (2021). Efficacy and cost-benefit of onsite contraceptive services with and without incentives among women with opioid use disorder at high risk for unintended pregnancy: a randomized clinical trial. JAMA Psychiat.

[29] Martin CE, Han JJ, Serio-Chapman C, Chaulk P, Terplan M (2014). Injectable contraceptive continuation among female exotic dancers seeking mobile reproductive health services. J Health Care Poor Underserved.

[30] Nall M, O’Connor S, Hopper T, Peterson H, Mahajan B (2019). Community women and reproductive autonomy: building an infrastructure for long-acting reversible contraception (LARC) services in a mobile health clinic. J Health Care Poor Underserved.

[31] Jacobstein R, Curtis C, Spieler J, Radloff S (2013). Meeting the need for modern contraception: Effective solutions to a pressing global challenge. Int J Gynecol Obstet.

[32] Moore E, Han J, Serio-Chapman C, Mobley C, Watson C, Terplan M (2012). Contraception and clean needles: Feasibility of combining mobile reproductive health and needle exchange services for female exotic dancers. Am J Public Health.

[33] Curtis KM, Jatlaoui TC, Tepper NK, et al. US selected practice recommendations for contraceptive use, 2016. MMWR Recomm Rep. 2016. 10.15585/mmwr.rr6504a1. 10.15585/mmwr.rr6504a1 27467319

